# Diagnostic Methods for and Clinical Pictures of Polyomavirus Primary Infections in Children, Finland 

**DOI:** 10.3201/eid2004.131015

**Published:** 2014-04

**Authors:** Tingting Chen, Laura Tanner, Ville Simell, Lea Hedman, Marjaana Mäkinen, Mohammadreza Sadeghi, Riitta Veijola, Heikki Hyöty, Jorma Ilonen, Mikael Knip, Jorma Toppari, Olli Simell, Maria Söderlund-Venermo, Klaus Hedman

**Affiliations:** University of Helsinki, Helsinki, Finland (T. Chen, L. Hedman, M. Sadeghi, M. Knip, M. Söderlund-Venermo, K. Hedman);; Haartman Institute, Helsinki (T. Chen, L. Hedman, M. Sadeghi, M. Söderlund-Venermo, K. Hedman);; University of Turku, Turku, Finland (L. Tanner, V. Simell, M. Mäkinen, J. Ilonen, J.Toppari, O. Simell);; Hospital District of Southwest Finland, Turku (V. Simell, M. Mäkinen);; Helsinki University Central Hospital, Helsinki (L. Hedman, M. Knip, K. Hedman);; University of Oulu, Oulu, Finland (R. Veijola);; University of Tampere, Tampere, Finland (H. Hyöty);; Fimlab laboratories, Tampere (H. Hyöty);; University of Eastern Finland, Kuopio, Finland (J. Ilonen);; Tampere University Hospital, Tampere (M. Knip);; Folkhälsan Research Center, Helsinki (M. Knip)

**Keywords:** Merkel cell polyomavirus, trichodysplasia spinulosa-associated polyomavirus, childhood, primary infection, serodiagnostics, viruses, Finland

## Abstract

We used comprehensive serodiagnostic methods (IgM, IgG, and IgG avidity) and PCR to study Merkel cell polyomavirus and trichodysplasia spinulosa-associated polyomavirus infections in children observed from infancy to adolescence. Comparing seroconversion intervals with previous and subsequent intervals, we found that primary infections with these 2 viruses were asymptomatic in childhood.

Two novel human polyomaviruses can cause skin diseases that are predominant in immunosuppressed persons. Merkel cell polyomavirus (MCPyV) is associated with Merkel cell carcinoma, which is an uncommon aggressive skin cancer, and trichodysplasia spinulosa-associated polyomavirus (TSPyV) is associated with trichodysplasia spinulosa, which is a rare skin disorder (*1**–**3*). In contrast with the rarity of the diseases, serologic studies have shown that both of these polyomaviruses infect humans of all ages; their seroprevalence has been assessed at 60%–80% among adults (*4*–*6*). We have reported high rates of MCPyV and TSPyV seroconversion among young children, indicating that primary exposures to these viruses occur extensively in early life (*5*,*7*). We observed children from infancy to 13 years of age by using comprehensive diagnostic methods for MCPyV and TSPyV and investigated pediatric primary infections with these 2 viruses for clinical correlates.

## The Study

This retrospective study was conducted during January 2011–July 2013 on a subset of a prospective study in which children were enrolled at birth and observed until young adolescence (*8*). We observed 144 children born during 1995–2004, from whom final samples were obtained during 2004–2008. 

On average, 13 serum samples per child were obtained during the study period (Technical Appendix Table 1). At each follow-up visit, clinical symptoms or illnesses since the previous visit were recorded (*8*). The ethics committee of the Hospital District of Southwest Finland (www.vsshp.fi/en/) approved the study.

IgG enzyme immunoassays (EIA) for MCPyV and TSPyV were conducted as described, except that we omitted subtraction of antigen-free background in the MCPyV assay (*5*,*7*). The respective lower and higher EIA cutoff values for IgG absence and presence were 0.120 and 0.210 for MCPyV and 0.100 and 0.240 for TSPyV (*5*,*7*). IgM EIAs for these 2 human polyomaviruses were developed as for human bocavirus 1(HBoV1) (*8*). The cutoff values were 0.207 and 0.260 for MCPyV and 0.194 and 0.240 for TSPyV. The IgG avidity assays were conducted as for HBoV1 (method A [*9*]). The respective cutoff values for low and high avidity were 15% and 25%.

Serum samples obtained during the final examination of each child were screened for MCPyV IgG and TSPyV IgG. Previous samples had not been tested. The children whose final samples lacked virus IgG were considered to be IgG negative; those whose final samples showed virus IgG were considered to have seroconverted. Each previous serum sample for each child who seroconverted was analyzed for IgG and IgM to identify the time period of seroconversion. Serum samples collected immediately before, at, and after the IgG seroconversion were examined for viral DNA; the seroconversion sample, the subsequent sample, and the final sample were examined for IgG avidity.

Of the 144 children, 45 (31%) showed IgG seroconversion for MCPyV and 39 (27%) for TSPyV. Before they were 1 year of age, 4 children showed IgG seroconversion for MCPyV at 0.68–0.94 year of age, 1 child showed seroconversion for TSPyV at 0.80 year, and another child showed MCPyV IgG, IgM, and low avidity of IgG in the first sample, which was collected at 0.63 year (Technical Appendix Table 2). None of these children who seroconverted early in life showed maternal IgG to the corresponding virus. Comparing participants at 0–1 year of age with those at 9–13 years, the seroprevalence for MCPyV caused by acquired infections rose from 3.4% to 65% and for TSPyV, from 0.7% to 53%. Seroconversions for each virus continued throughout the study (Figure).

**Figure:**
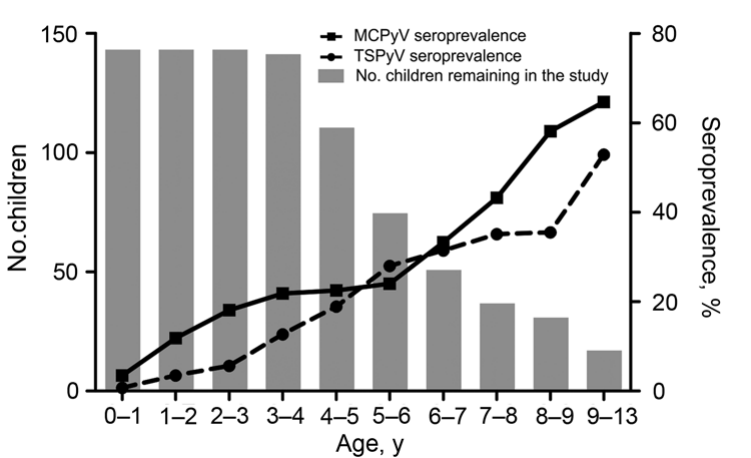
Seroprevalence related to polyomavirus primary infections in children in Finland during follow-up, January 2011–July 2013. Seroprevalence was calculated by the formula: Seroprevalence = (no. seropositive children remaining in the study at each age category) × 100.

Of the 45 children who seroconverted for MCPyV, 28 (62%) showed additional markers of primary infection at the time of IgG seroconversion: IgM was present in 15 (33%), and low avidity of IgG was detected in 23 (51%). Of the 39 TSPyV–seroconverted children, 32 (82%) showed corresponding markers: IgM in 30 (67%) and low avidity of IgG in 13 (29%). Samples did not show MCPyV viremia at or flanking the seroconversion, and TSPyV viremia was observed at low quantity (<10^4^ copies/mL) in the samples of 2 who seroconverted. Except 4 seroconverters for MCPyV and 1 for TSPyV, all showed long-term maturation of IgG avidity to the corresponding virus.

Maternal IgG showed in 10 (22%) of the 45 who seroconverted for MCPyV and in 12 (31%) of the 39 for TSPyV at sampling ages of 0.23–0.62 year; these maternal IgGs were no longer discernable at 0.49–1.07 year. After the first year of life, the age at seroconversion with either virus did not appear to correlate with the presence or absence of maternal antibodies.

To determine clinical correlates of MCPyV and TSPyV primary infections, all infection-related symptoms and illnesses during the seroconversion interval were compared with those during the previous or subsequent interval for each patient who seroconverted (Table). Infection-related symptoms during the seroconversion interval were reported for 73% of children who seroconverted for MCPyV and for 82% of those who seroconverted for TSPyV. The occurrences of symptoms, however, were not notably different from those during the previous or subsequent intervals. Exanthema was reported in 7 (15.9%) children at the MCPyV seroconversion interval and in 1 child in each of the adjacent periods (2.2% before, 2.3% after). However, the differences were not statistically significant (p = 0.0703).

**Table:** Infection-related signs and symptoms during MCPyV and TSPyV seroconversions compared with previous and subsequent intervals for asymptomatic polyomavirus infections in children, Finland, January 2011–July 2013*

Virus sign/symptom	Interval, no. (%)		Previous interval			Subsequent interval	
			No. (%)	p value		No. (%)	p value
MCPyV seroconversion, n = 45							
URTI	14 (31.8)		10 (22.2)	0.4545		18 (41.9)	0.3018
LRTI	3 (6.8)		2 (4.4)	1.0000		3 (7.0)	1.0000
Fever without RTI	2 (6.8)		2 (4.4)	1.0000		3 (7.0)	0.6250
Gastroenteritis	9 (20.5)		9 (20.0)	1.0000		11 (25.6)	0.5488
Acute tonsillitis	1 (2.3)		0	ND		1 (2.3)	1.0000
Acute otitis media	10 (22.7)		11 (24.4)	1.0000		13 (30.2)	0.6291
Acute conjunctivitis	2 (4.5)		1 (2.2)	1.0000		1 (2.3)	1.0000
Exanthema	7 (15.9)		1 (2.2)	0.0703		1 (2.3)	0.0703
Acute sinusitis	2 (4.5)		0	ND		1 (2.3)	1.0000
Total	32 (72.7)		26 (57.8)	0.2632		31 (72.1)	1.0000
No data	1		0	NA		2	NA
TSPyV seroconversion, n = 39							
URTI	14 (35.9)		9 (23.1)	0.3593		13 (35.1)	1.0000
LRTI	3 (7.7)		3 (7.7)	1.0000		2 (5.4)	1.0000
Fever without RTI	2 (5.1)		5 (12.8)	0.3750		4 (10.8)	0.6875
Gastroenteritis	10 (25.6)		12 (30.8)	0.7744		5 (13.5)	0.3877
Acute tonsillitis	1 (2.6)		1 (2.6)	1.0000		2 (5.4)	1.0000
Acute otitis media	8 (20.5)		12 (30.8)	0.3877		8 (21.6)	1.0000
Acute conjunctivitis	4 (10.3)		5 (12.8)	1.0000		3 (8.1)	1.0000
Exanthema	2 (5.1)		3 (7.7)	1.0000		1 (2.7)	1.0000
Acute sinusitis	3 (7.7)		2 (5.1)	1.0000		2 (5.4)	ND
Total	32 (82.1)		30 (76.9)	0.7266		30 (81.1)	1.0000
No data	0		0	NA		2	NA
*Liddell exact test was used; p<0.05 was considered significant. MCPyV, Merkel cell polyomavirus; TSPyV, trichodysplasia spinulosa-associated polyomavirus; LRTI, lower respiratory tract infection; URTI, upper respiratory tract infection; ND, no data; NA, not applicable.							

## Conclusions

The seroprevalence of MCPyV and TSPyV among children <13 years of age increased because of acquired infections, a finding consistent with reports that primary infections with these 2 viruses are ubiquitous in childhood (*5*,*7*). The seroprevalence of these viruses had not reached a plateau by the end of study; thus, some infections are expected to occur later than 13 years of age. We expect that some children have become infected with these viruses after exiting the study.

For both viruses, prevalence of maternal antibodies during infancy was high, supporting recent findings by Martel-Jantin et al. (*10*). Although the age of the child at seroconversion did not appear to correlate with the presence of maternal antibodies, these antibodies were absent from children who showed viral infection during infancy, raising the possibility that maternal immunity may protect infants from infection by these viruses. However, the prevalence of MCPyV infection among infants was higher than that of TSPyV, possibly caused by MCPyV shedding from parental skin (*10*–*12*).

We did not observe any symptom associated with the time of seroconversion for these 2 viruses, which implies that primary exposures to these viruses during childhood cause no symptoms. When studying MCPyV infections in adult men who seroconverted, Tolstov et al. also found no clinical associations (*13*). Their observations and ours in the current study of MCPyV and TSPyV support the common notion that the prototypic human PyVs, BKPyV and JCPyV, also establish persistence without initial signs (*14*,*15*).

Exanthema, a common sign of skin infection, did not appear infrequently during MCPyV seroconversion; however, the rate did not reach statistical significance (p = 0.0703), which might be related to our limited cohort size. We cannot rule out that data for some transient skin-related signs may not have been captured during periodic patient interviews. Last, we do not believe that our study represents polyomavirus infections during childhood worldwide. Larger studies of more geographically diverse populations are needed to determine whether primary infection with MCPyV or TSPyV is always asymptomatic.

Although IgM and low-avidity IgG were observed in more than half of the seroconversion samples, the frequency of viremia was extremely low, indicating serodiagnostics as the strategy of choice in diagnosing primary infections with MCPyV and TSPyV. Our study suggests that these ubiquitous polyomaviruses circulate silently among children.

Technical Appendix Distribution of outcomes of serodiagnostic methods, Finland, January 2011–July 2013.
