# Supplementary material for: Diagnostic Methods for and Clinical Pictures of Polyomavirus Primary Infections in Children, Finland
Source: Emerg Infect Dis. 2014 Apr;20(4):689–92. doi: 10.3201/eid2004.131015 (PMC3966366; doi:10.3201/eid2004.131015)
Supplement: Technical Appendix — Distribution of outcomes of serodiagnostic methods, Finland, January 2011–July 2013. [file 13-1015-Techapp-s1.pdf]

Article DOI: 10.3201/eid2004.131015

Diagnostic Methods for and Clinical Pictures of Polyomavirus Primary Infections in Children, Finland

Technical Appendix

Distribution of Outcomes of Serodiagnostic Methods

Technical Appendix Table1. Distribution of outcomes of serodiagnostic methods used to detect polyomavirus primary infections in children, Finland, January 2011–July 2013

		Seroconverted participants, no.		
Sample parameters	All participants, N = 144	MCPyV, n = 45	TSPyV, n = 39	Total, n = 74
Serum samples, no. per child	1,929	665	583	1,081
Mean	13	15	15	15
Median	12	15	13	13
Range	8–27	8–24	8–27	8–27
Age at sampling initiation, y				
Mean	0.36	0.36	0.37	0.37
Median	0.31	0.32	0.30	0.32
Range	0.20–0.91	0.22–0.79	0.20–0.60	0.20–0.79
Age at sampling end, y				
Mean	5.77	6.50	6.67	6.48
Median	5.07	6.03	6.03	5.98
Range	2.56–12.48	3.46–10.51	3.46–12.48	3.46–12.48
Sampling interval, age $\leq$2 y, mo.				
Mean	3.65	3.74	3.68	3.73
Median	3.23	3.27	3.23	3.25
Range	1.5–16.13	2.03–16.13	2.03–8.53	2.03–16.13
Sampling interval, age >2 y, mo.				
Mean	6.35	6.24	6.41	6.35
Median	6.03	6.00	6.10	6.03
Range	2.13–28.3	2.77–18.20	2.47–19.40	2.47–19.40

Technical Appendix Table 2. Serologic data of children showing or TSPyV primary infections before 1 year of age, Finland, January 2011–July 2013 *

Subject no.	Sample no.	Age, y	IgM	IgG	IgG avidity
MCPyV					
#47	1	0.27	0.010	0.045	ND
	2	0.87	0.021	2.003	24.1
#118	1	0.41	0.009	0.012	ND
	2	0.80	0.251	2.697	19.4
#147	1	0.42	0.064	0.009	ND
	2	0.68	0.026	0.516	14.9
#186	1	0.48	0.009	0.013	ND
	2	0.74	0.482	0.021	ND
	3	0.94	0.222	1.386	13.5
#167	1	0.63	0.320	0.568	4.5
	2	1.10	0.054	0.652	26.5
TSPyV					
#157	2	0.49	0.023	0.010	ND
	3	0.80	0.071	2.317	54.7

*MCPyV, Merkel cell polyomavirus; TSPyV, trichodysplasia spinulosa-associated polyomavirus; ND, not detected.
